# SNP (–617C>A) in ARE-Like Loci of the NRF2 Gene: A New Biomarker for Prognosis of Lung Adenocarcinoma in Japanese Non-Smoking Women

**DOI:** 10.1371/journal.pone.0073794

**Published:** 2013-09-11

**Authors:** Yasuko Okano, Uru Nezu, Yasuaki Enokida, Ming Ta Michael Lee, Hiroko Kinoshita, Alexander Lezhava, Yoshihide Hayashizaki, Satoshi Morita, Masataka Taguri, Yasushi Ichikawa, Takeshi Kaneko, Yutaka Natsumeda, Tomoyuki Yokose, Haruhiko Nakayama, Yohei Miyagi, Toshihisa Ishikawa

**Affiliations:** 1 Omics Science Center, RIKEN Yokohama Institute, Yokohama, Japan; 2 Department of Clinical Oncology, Yokohama City University Graduate School of Medicine, Yokohama, Japan; 3 Department of Clinical Pharmacology and Therapeutics, Graduate School of Medicine, University of the Ryukyus, Okinawa, Japan; 4 Division of Thoracic and Visceral Organ Surgery, Gunma University Graduate School of Medicine, Maehashi, Japan; 5 Laboratory for International Alliance on Genomic Research, RIKEN Center for Integrative Medical Sciences, Yokohama, Japan; 6 Institute of Biomedical Sciences, Academia Sinica, Taipei, Taiwan; 7 Department of Biostatistics and Epidemiology, Yokohama City University Medical Center, Yokohama, Japan; 8 Respiratory Disease Center, Yokohama City University Medical Center, Yokohama, Japan; 9 Department of Clinical Research, Yokohama City University Graduate School of Medicine, Yokohama, Japan; 10 Department of Pathology, Kanagawa Cancer Center Research Institute, Yokohama, Japan; 11 Department of Thoracic Surgery, Kanagawa Cancer Center Research Institute, Yokohama, Japan; 12 Kanagawa Cancer Center Research Institute, Yokohama, Japan; National Taiwan University, Taiwan

## Abstract

**Purpose:**

The transcription factor NRF2 plays a pivotal role in protecting normal cells from external toxic challenges and oxidative stress, whereas it can also endow cancer cells resistance to anticancer drugs. At present little information is available about the genetic polymorphisms of the *NRF2* gene and their clinical relevance. We aimed to investigate the single nucleotide polymorphisms in the *NRF2* gene as a prognostic biomarker in lung cancer.

**Experimental Design:**

We prepared genomic DNA samples from 387 Japanese patients with primary lung cancer and detected SNP (c.–617C>A; rs6721961) in the ARE-like loci of the human *NRF2* gene by the rapid genetic testing method we developed in this study. We then analyzed the association between the SNP in the *NRF2* gene and patients’ overall survival.

**Results:**

Patients harboring wild-type (WT) homozygous (c.–617C/C), SNP heterozygous (c.–617C/A), and SNP homozygous (c.–617A/A) alleles numbered 216 (55.8%), 147 (38.0%), and 24 (6.2%), respectively. Multivariate logistic regression models revealed that SNP homozygote (c.–617A/A) was significantly related to gender. Its frequency was four-fold higher in female patients than in males (10.8% female vs 2.7% male) and was associated with female non-smokers with adenocarcinoma. Interestingly, lung cancer patients carrying *NRF2* SNP homozygous alleles (c.–617A/A) and the 309T (WT) allele in the *MDM2* gene exhibited remarkable survival over 1,700 days after surgical operation (log-rank p = 0.021).

**Conclusion:**

SNP homozygous (c.–617A/A) alleles in the *NRF2* gene are associated with female non-smokers with adenocarcinoma and regarded as a prognostic biomarker for assessing overall survival of patients with lung adenocarcinoma.

## Introduction

Lung cancer is the leading cause of cancer-related death in many industrial countries. It is classified into two major types, namely, small-cell lung carcinoma (SCLC) and non-small-cell lung carcinoma (NSCLC). While long-term exposure to cigarette smoke is the most common cause of lung cancer (80–90% of lung cancers), non-smokers account for 10–15% of lung cancer cases, which are often attributed to a combination of genetic and environmental factors [1]–[3]. The transcription factor NF-E2-related factor 2 (NRF2) is known to control cellular adaptation/protection to reactive oxygen species and electrophiles by inducing antioxidation and detoxification genes [4]–[6] as well as mediate cancer cell proliferation and drug resistance [7]–[12]. We have undertaken the present study to examine the clinical impact of the *NRF2* gene and its genetic polymorphisms on the risk and prognosis of lung cancer.

NRF2 is a “cap‘n’collar” basic region-leucine zipper (CNC-bZip) transcription factor and plays a pivotal role in the induction of antioxidant response element (ARE)-regulated genes [4]–[13]. Under non-stressed conditions, NRF2 protein is associated with Kelch-like ECH associating protein 1 (*KEAP1*) [14]. KEAP1 is known to be a negative regulator of NRF2 by retrieving it in the cytoplasm. Oxidative stress and/or electrophilic attack lead to the dissociation of NRF2 from KEAP1 and thereby the NRF2 protein is translocated into the nucleus. NRF2 together with small multiple alignment format (MAF) sequences binds to ARE sequences [15]. Many genes encoding detoxifying and antioxidant enzymes have been found to be regulated by NRF2 [4]–[6], [15]–[18]. It has recently been reported that NRF2 contributes to malignant phenotypes of cancer cells *in vitro*, including aggressive cell proliferation, drug resistance, and metabolic re-programming [7]–[11], [19], [20]. In this context, the *NRF2* gene is considered to play split roles, for example, in the protection of normal cells and progression of cancer malignancy.

In 2004, Yamamoto and colleagues first reported the structure of the *NRF2* gene and found three SNPs (−653A>G, −651G>A, and –617C>A) and one triplet repeat polymorphism in its regulatory region [21]. Three years later, Marzec *et al.* examined the impact of those SNPs on the regulation of *NRF2* gene expression [22]. In transient transfection assays, they found that the –617C>A SNP significantly affects basal NRF2 protein levels and its function *in vitro* [22]. SNP –617C>A was found to be associated with a higher risk of oxidant-induced acute lung injury in humans [22]. It has been reported that a SNP (c.–617C>A) in the ARE-like loci of the human *NRF2* gene is important for self induction of the *NRF2* gene. *NRF2* regulates the transcription of numerous phase II drug-metabolizing enzymes and phase III drug-transporters (*e.g.*, ABCC1, ABCC2, ABCC4, and ABCG2) in response to oxidative stress *via* direct binding to the ARE sequences in those target genes [23]–[26]. At present, however, little information is available as to the clinical impact of genetic polymorphisms of the *NRF2* gene and the prognosis of lung cancer.

To gain insight into the genetic polymorphisms of the *NRF2* gene, we have developed rapid genotyping primer sets by utilizing the SmartAmp method, an isothermal DNA amplification process [27], [28]. Among a total of 387 lung cancer patients, we found that SNP (c.–617C>A) in the *NRF2* gene is a prognostic biomarker for assessing the gender (female)-related risk of lung adenocarcinomas in the Japanese non-smoking sub-population of lung cancer patients. The epidermal growth factor receptor (*EGFR*) gene was frequently mutated in those female patients harboring the SNP homozygous SNP allele (–617A/A), suggesting a potential link between the SNP homozygote (–617A/A) and *EGFR* gene mutations. Furthermore, NRF2 reportedly regulates expression of the *MDM2* gene that encodes a negative regulator of p53, and this study shows that lung cancer patients with homozygous SNP alleles (–617A/A) in the *NRF2* gene and the 309T (WT) allele in the *MDM2* gene had markedly better overall survival. This is the first report providing clinical evidence that homozygous SNP (–617A/A), as one of the intrinsic genetic polymorphisms in the *NRF2* gene, is associated with the overall survival of lung cancer patients. Our clinical research data strongly suggest that the SNP homozygous allele (–617A/A) is a useful biomarker for clinical diagnosis.

## Results

### Clinicopathological Characterization

The clinicopathological characterization data for the 387 primary lung cancer patients are summarized in Table 1. The patient population comprised 221 men and 166 women, with an overall mean age of 66 years (range 35 to 87 years). The histological type of lung cancer was determined according to the protocol of the third World Health Organization/International Association for the Study of Lung Cancer Classifications [29]. Among the lung cancer patients, 298 were classified as having adenocarcinomas and 89 non-adenocarcinomas. The p-stage was determined by pathological examination of surgical specimens for 376 patients, and their tumours were staged according to the tumor nodes metastasis (TNM) classification of malignant tumours: 292, 46, 35, and 3 patients were respectively classified into stages I, II, III, and IV. For the remaining 11 patients, the p-stage could not be determined. The smoking history was obtained from each lung cancer patient at Kanagawa Cancer Center Research Institute: 154 patients had no smoking history, whereas 233 patients were smokers.

**Table 1:** Clinicopathological characterization of primary lung cancer patients.

Variable	No. of patients	(%)
Gender		
Male	221	(57.1)
Female	166	(42.9)
Age (years old)		
≤50	25	(6.4)
>50	362	(93.5)
Histopathology		
Adeno	298	(77.0)
Non-adeno	89	(23.0)
Smoking		
Non-smoker	154	(39.8)
Smoker	233	(60.2)
p-Stage		
I	292	(75.4)
II	46	(11.9)
III	35	(9.0)
IV	3	(0.8)
Undetermined	11	(2.8)

Ages of all patients, 66.4±9.9 (mean±S.D.).

Abbreviation: Adeno, adenocarcinoma.

### Preparation of Genotyping Primers for Detection SNP (c.– 617 C>A) in *NRF2* Gene

The *NRF2* gene is located on the negative strand of genomic DNA at q31.2 of chromosome 2. To create the template for preparation of genotyping primers, we synthesized double stranded DNA encoding a 424-bp region of nt.178129735 to nt.178130158, including the SNP (c. –617 C>A) in the *NRF2* gene by means of PCR with human genomic DNA. PCR primers used for the DNA synthesis were GACCACTCTCCGACCTAAAGG (forward) and CGAGATAAAGAGTTGTTTGCGAA (reverse). The resulting PCR product was then inserted into the TA-cloning site of the pGEM®-T Easy Vector (Promega, Madison, WI, USA). The vector was amplified in JM109 High Efficiency Competent Cells (Promega), and vector DNA was purified by using the GeneGET™ Plasmid Miniprep Kit (Fermentas, Thermo Fisher Scientific Inc., Waltham, MA, USA). The sequence of the inserted DNA was analyzed with a laser-based automated DNA sequencer (ABI PRISM 3100 DNA Analyzer, Applied Biosystems Ltd., Tokyo, Japan). We designed a number of SNP-typing primer candidates and repeatedly tested them by using the vector-inserted DNA as a template until we obtained the best primer set.

**Figure 1:**
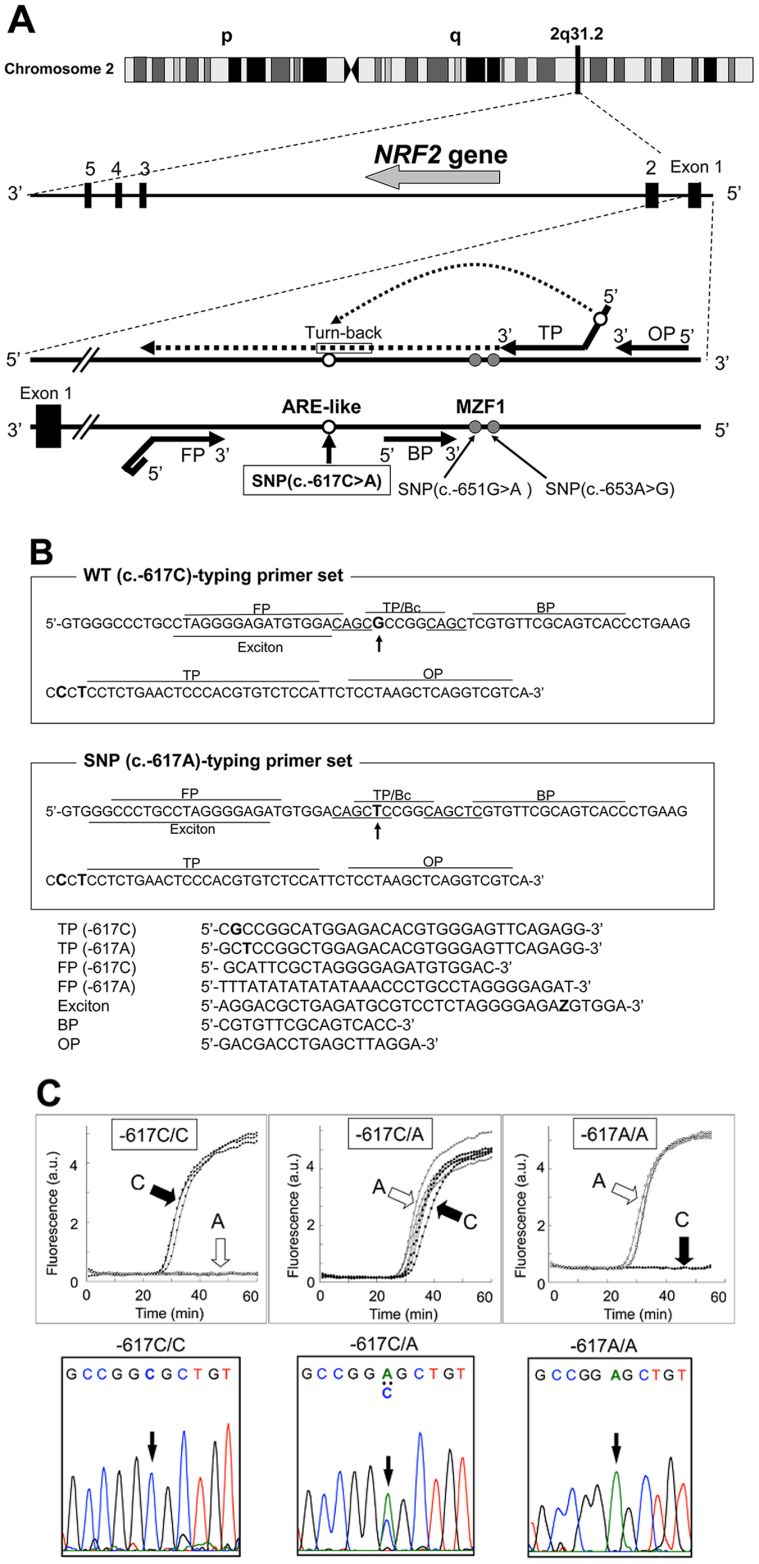
SmartAmp-based detection of SNP (c.–617C>A) in the *NRF2* gene. SNP (c.–617C>A) resides in the promoter region of the *NRF2* gene on chromosome 2q31.2. Panel **A** presents a schematic illustration of annealing sites of the TP, FP, and OP primers. Panel **B** shows cDNA encoding a partial sequence of the *NRF2* gene and primer annealing sites. Panel **C** depicts the results of SNP detection. a.u. = arbitrary unit.

The schematic illustration in Figure 1A shows the annealing sites of the best primer set, which comprises four different primers, *i.e*., TP, OP, FP, and BP. The TP primer was designed such that its turn-back region can discriminate the nucleotide of C or A at c.–617 in the negative DNA strand extended from the 3′-end of the primer (see Figure 1A). Furthermore, two SNPs, c.−651G>A and c.653A>G, were not included in the annealing sites of the four primers. Figure 1B depicts the sequences of primers in the WT (–617C)-typing and SNP (–617)-typing sets. Exciton dye was linked to thymine in the FP primer as symbolized by “Z” in the lower panel of Figure 1B.

Figure 1C shows the time courses of genotyping reactions as functions of fluorescence intensity. By using the genotyping primer sets, we could detect the WT homozygote (–617C/C), WT/SNP heterozygote (–617C/A), and SNP homozygote (–617A/A) in genomic DNA samples. These results were verified by DNA sequence analysis. Namely, we performed sequencing analysis for determination of the WT homozygote (–617C/C), WT/SNP heterozygote (–617C/A), and SNP homozygote (–617A/A) in those genomic DNA samples. We tested 24 samples for each group (i.e., C/C, C/A, and A/A) and confirmed the 100% accordance between the SmartAmp method and the DNA sequencing analysis method.

### Detection of SNP (c.–617 C>A) in the *NRF2* Gene in Lung Cancer Patients

Using the genomic DNA samples prepared from a total of 387 lung cancer patients, we have detected WT and SNP alleles in the *NRF2* gene by the rapid genotyping method described above. Table 2 summarizes these results, showing that 216, 147, and 24 patients could be typed as WT homozygote (–617C/C), WT/SNP heterozygote (–617C/A), and SNP homozygote (–617A/A), respectively. Accordingly, the allele frequency was calculated to be 74.8% and 25.2% for WT (c.–617C) and SNP (c.–617A), respectively, when both male and female patients were grouped together. It is of interest to note, however, that the allele frequency of SNP (c.–617A) was 28.6% for female patients, compared with 22.6% for male patients. Indeed, 18 female patients carried the SNP homozygote (–617A/A), a number three-fold higher than that of male patients. The ratio of homozygous SNP (–617A/A) was 10.8% for female patients, about four-fold higher than the 2.7% found in males (Table 2; *P = *0.004). In contrast, the ratios of WT homozygote (–617C/C) and heterozygote (–617 C/A) were moderately higher in male patients than in female patients (Table 2). On the other hand, with respect to smoking experience, the ratio of homozygous SNP (–617A/A) was 10.4% in the non-smoker sub-population, being about three times (*P = *0.021) higher than the ratio (4.5%) observed in the smokers (Table 2).

**Table 2:** Classification of primary lung cancer patients with respect to *NRF2* genotypes, gender, and histopathology.

	*NRF2* gene SNP (–617)					
	C/C		C/A		A/A	*P*-value*
Patients	216 (55.8)		147 (38.0)		24 (6.2)	
Gender*						
Male	127 (57.5)		88 (39.8)		6 (2.7)	
Female	89 (53.6)		59 (35.5)		18 (10.8)	0.004
Histopathology						
Adeno	164 (55.0)		114 (38.3)		20 (6.7)	
Non-adeno	52 (58.4)		33 (37.0)		4 (4.5)	0.687
Smoking behavior*						
Smoker	133 (58.4)		92 (37.0)		8 (4.5)	
Non-smoker	83 (53.9)		55 (35.7)		16 (10.4)	0.021
p-Stage						
I	156 (53.4)	114 (39.0)		22 (7.5)		
II	28 (60.9)	16 (34.8)		2 (4.3)		
III	23 (65.7)	12 (34.3)		0 (0)		
IV	1 (33.3)	2 (66.7)		0 (0)		0.459

The number of patients (%).

* P- values were calculated by Fisher’s exact test.

Abbreviation: Adeno, adenocarcinoma.

By multivariate analysis, the SNP homozygote (–617A/A) in the *NRF2* gene was found to be independently associated with gender (Table 3). Among a total of 20 adenocarcinoma patients (females+males) carrying the SNP homozygote (–617A/A), 16 patients were females who had no cigarette-smoking experience (Table 4). In contrast, there were no male adenocarcinoma patients in the sub-group of non-smokers carrying the SNP homozygote (–617A/A) (Table 4). These results demonstrate a marked gender difference in terms of non-smoking patients carrying the SNP homozygote (–617A/A).

**Table 3:** Logistic regression analysis for evaluation of the association among homozygous SNP alleles (–617A/A) in the *NRF2* gene and gender/smoking experience of lung cancer patients.

Variable	*P*	Odds Ratio	95% CI
Gender	0.041	3.48	1.05 to 11.51
Smoking	0.463	0.66	0.22 to 2.00

Abbreviation: CI, confidence interval.

Gender code: 1 = female; 0 = male.

Smoking experience code: 1 = smoker; 0 = non-smoker.

The multivariate logistic regression analysis was performed under two categories, *i.e.*, the gender (female and male) and the smoking experience (smoker and non-smoker).

**Table 4:** Classification of primary lung cancer patients with respect to *NRF2* genotypes, smoking behavior, adenocarcinoma, and gender.

		NRF2 gene SNP (–617)		
	C/C	C/A	A/A	*P*-value*
Patients (M+F)	216	147	24	
Smoking behavior				
Smoker (M)	114	75	6	
Smoker (F)	19	17	2	
Non-smoker (M)	13	13	0	
Non-smoker (F)	70	42	16	0.014
Adenocarcinoma				
Smoker (M)	75	48	4	
Smoker (F)	15	16	0	
Non-smoker (M)	11	13	0	
Non-smoker (F)	63	37	16	0.003

* *P*-values were calculated by Fisher’s exact test.

Abbreviation: M, male; F, female.

To gain more insight into the gender difference among 24 patients homozygous for the SNP (c.–617A/A), we have analyzed the genetic polymorphisms of human CYP2A6*4 (whole gene deletion) and the numbers of (GT)_n_ repeats in the *HO-1* gene 5′-flanking region. These data are summarized in Table 5. CYP2A6*4/*4 (whole gene deletion) was found in only one female patient in this subgroup (Table 5), whereas among 387 lung cancer patients, CYP2A6*4/*4 was detected in seven patients (1.8%) with lung adenocarcinoma. The number of (GT)_n_ repeats in the *HO-1* gene 5′-flanking region varied greatly (14 to 34 repeats) among these 24 patients.

**Table 5:** Clinicopathological profiling of 24 patients harboring homozygous SNP alleles (–617A/A) in the *NRF2* gene.

Case	Histology	p stage	Age	Gender	smoker	(GT)n repeats	CYP2A6	EGFR mutation	Gefitinib therapy
1	Ad	IIA	74	F	non-smoker	19,30	Wt	Exon 21	Yes
2	Ad	IA	53	F	non-smoker	23	*4/*4	Exon 19	–
3	Ad	IB	70	F	non-smoker	24	Wt	Exon 21	–
4	Mix	IB	63	F	non-smoker	34	Wt	Exon 21	–
†5	Ad	IB	61	F	non-smoker	23	Wt	Exon 19	–
6	Ad	IB	72	F	non-smoker	22	Wt	Exon 21	–
7	Ad	IA	40	F	non-smoker	19	Wt	Exon 19	–
8	Ad	IA	71	F	non-smoker	17	Wt	Exon 21	–
9	Ad	IA	45	F	non-smoker	30	Wt	Exon 21	–
10	Ad	IA	74	F	non-smoker	30	Wt	Exon 21	–
11	Ad	IA	73	F	non-smoker	22	Wt	Exon 19	–
12	Ad	IA	69	F	non-smoker	22	Wt	Exon 21	–
13	Ad	IA	62	F	non-smoker	28	Wt	None	–
14	Ad	IA	73	F	non-smoker	14	Wt	Exon 19	–
15	Ad	IA	63	F	non-smoker	15	Wt	Exon 19	–
16	Ad	IIA	73	F	non-smoker	30	Wt	None	Yes
17	Sq	IA	72	F	smoker	20	Wt	None	–
18	Ple	IA	75	F	smoker	29	Wt	None	–
19	Ad	IA	74	M	smoker	23,27	Wt	None	–
20	Sq	IA	78	M	smoker	23	Wt	None	–
21	Ad	IA	65	M	smoker	30	Wt	Exon 21	–
22	Sq	IB	75	M	smoker	20	Wt	None	–
23	Ad	IA	77	M	smoker	28,31	Wt	Exon 21	–
24	Ad	IA	80	M	smoker	29,30	Wt	Exon 19	–

Abbreviation: Ad, adenocarcinoma; Mix, adenocarcinoma and squamous cell carcinoma; Ple, pleomorphic carcinoma; Sq, squamous cell carcinoma; F, female; M, male; Wt, wild type.

† Patient (case 5) died because of primary pancreatic cancer.

Interestingly, as shown in Table 5, either exon 19 or exon 21 of the *EGFR* gene was frequently mutated in female patients who were non-smokers and had homozygous SNP alleles (–617A/A). Two of those patients were diagnosed as p-stage IIA (cases 1 and 16). They were treated first surgically and then with gefitinib. These patients had no relapse over 1879 days (case 1) and 939 days (case 16).

### Association of SNP (c.–617 C>A) in the *NRF2* Gene with Overall Survival of Lung Cancer Patients

**Figure 2:**
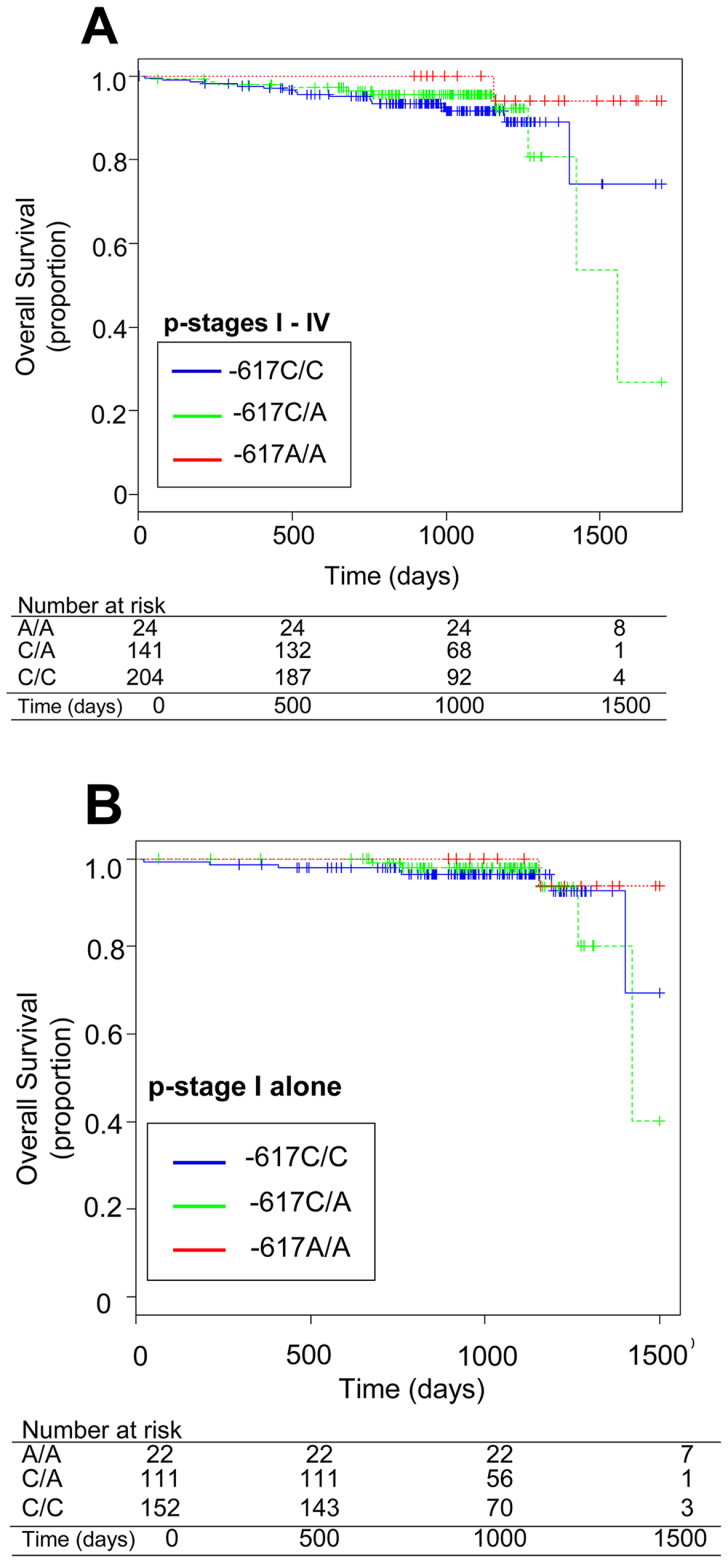
Kaplan-Meier plots showing the overall survival of patients harboring the WT homozygote (–617C/C), WT/SNP heterozygote (–617C/A), or SNP homozygote (–617A/A) in the *NRF2* gene. Patients with p-stages I to IV (**A**) and p-stage I only NSCLC (**B**). The number of patients at times 0, 500, 1000, or 1500 days after surgical operation is described along with genotypes of the *NRF2* gene.

We investigated a potential association between SNP (c.–617 C>A) in the *NRF2* gene and the overall survival of lung cancer patients, since we could obtain follow-up information on 369 patients among the total of 387 lung cancer patients over 1,700 days after surgical operation at Kanagawa Cancer Center. A survival Kaplan-Mayer plot is shown in Figure 2A. Our univariate analysis has revealed that lung cancer patients (p-stages I to IV) carrying homozygous SNP alleles (–617A/A) in the *NRF2* gene experienced significantly better overall survival, as compared with patients with heterozygous alleles (c.–617C/A) (log-rank *P = *0.021). In contrast, no association was found between patients with homozygous WT alleles (–617C/C) and those with heterozygous alleles (c.–617C/A) (Figure 2A). It is important to mention that one female patient with the homozygous SNP (–617A/A) (case 5 in Table 5) died due to primary pancreatic cancer during the follow-up study.

We further analyzed the associations between the *NRF2* genotypes and patients’ overall survival in the p-stage I of non-small cell lung cancer (NSCLC) including adenocarcinoma, squamous cell cancer, and large cell cancer. In the case of stage I, patients (n = 285) were treated by surgical excision of the tumor, with no follow-up treatment by adjuvant therapy or chemotherapy. Nonetheless, patients harboring homozygous alleles (–617A/A) in the *NRF2* gene exhibited the best record of overall survival among the members of these three different allele groups (Figure 2B).

### Potential Link between *NRF2* and *MDM2* Genotypes

NRF2 reportedly regulates expression of the *MDM2* gene that encodes a crucial negative regulator of p53. To gain insight into a potential link between *MDM2* and *NRF2* genotypes, we have analyzed the SNP (c.309T>G) in the *MDM2* gene as well as the SNP (–617C>A) in the *NRF2* gene using the genomic DNA samples from lung cancer patients. Table 6 summarizes the corresponding results, where the number of patients harboring T/T, T/G, or G/G genotype in the *MDM2* gene has been given for each genotype of the *NRF2* gene (*i.e*., –617C/C, C/A. or A/A). In the genotype groups of –617C/C and –617C/A, the 309G (SNP) allele frequency was 0.606 and 0.541, respectively. In contrast, the SNP allele frequency was found to be markedly lower (0.333) in the genotype group of *NRF2* –617A/A. In the case of adenocarcinoma, female patients harboring 309T/T, T/G, and G/G genotype in the *MDM2* gene were 7, 8, and 1, respectively (Table S1); most patients were harboring the 309T (WT) allele in the *MDM2* gene.

**Table 6:** Classification of primary lung cancer patients with respect to genotypes of *NRF2* and *MDM2* genes.

	NRF2 (–617)		
	C/C	C/A	A/A
Patients (N)	216	147	24
MDM2 (c.309)	N (%)	N (%)	N (%)
T/T	35 (16.2)	36 (24.5)	11 (45.8)
T/G	100 (46.3)	63 (42.9)	10 (41.7)
G/G	81 (37.5)	48 (32.6)	3 (12.5)

N, the number of patients; % in parentheses.

## Discussion

### SNP (c.–617C>A) in the *NRF2* Gene and Female Non-smokers with Adenocarcinoma

Recent genome-wide association studies (GWAS) have identified several loci associated with lung cancer susceptibility in never-smoking women in Asia; they were, 5p15.33 (rs2736100) [30], 6p21.3 (rs3817963) [30], 3q28 (rs10937405 and rs4488809) [31], 10q25.2 (rs7086803) [32], 6q22.2 (rs9387478) [32], and 6p21.32 (rs2395185) [32].

In the present study, differing from those reports, we found that SNP (c.–617C>A; rs6721961) in the *NRF2* gene located on chromosome 2q31.2 is associated with Japanese non-smoking female patients with adenocarcinoma and their overall survival. While the allele frequency of SNP c.–617C>A in the *NRF2* gene was estimated to be 25.2%, non-smoking females harboring homozygous alleles (–617A/A) had a markedly higher incidence of adenocarcinoma (Table 4, Table 5), as compared with non-smoking males harboring the same genotype. In other words, the –617 C>A SNP in the ARE-like loci of the human *NRF2* gene seems to be associated with female non-smokers with adenocarcinoma. Furthermore, it is noteworthy that the *EGFR* gene was frequently mutated in female patients who were non-smokers and had homozygous SNP alleles (–617A/A) in the *NRF2* gene (Table 5), suggesting a potential link between the SNP homozygote (–617A/A) and *EGFR* gene mutations.

Recent studies have demonstrated that mutations in the tyrosine kinase domain of the EGFR are frequently found among non-smoker patients with NSCLC [33]. Approximately 90% of these mutations are exon 19 deletions or exon 21 L858R point mutations in the tyrosine kinase domain [34]. In the vast majority of cases, *EGFR* mutations are non-overlapping with other oncogenic mutations (e.g., *KRAS* mutations, *ALK* rearrangements) found in NSCLC [34]. A large randomized clinical study named the “IRESSA Pan-Asian Study (IPASS)” has reported that high rates of mutations in the *EGFR* gene were observed in female NSCLC patients without smoking experience [35]. A high incidence of *EGFR* gene mutations was reported in female non-smokers with adenocarcinoma of lung: 30–40% in East Asians, as compared with 15% in Caucasians [36]–[38]. Both *EGFR* gene mutations and homozygous SNP alleles (–617A/A) in the *NRF2* gene were frequently observed in Japanese female adenocarcinoma patients without smoking experience (Table 4). As shown in Table 7, ethnic group-dependent difference was observed in the *NRF2* genotype, where the frequency of the –617A allele is high in Japanese, Taiwanese, and Chinese populations. Thus, it is of great interest to investigate the link between the SNP homozygote (–617A/A) and *EGFR* gene mutations and to gain insight into the underlying molecular mechanism.

**Table 7:** Frequencies of wild type (–617C) and SNP (–617A) alleles in the *NRF2* gene among different ethnic groups.

	Allele frequency		NRF2 (–617)				
Ethnic group	C	A	C/C	C/A	A/A	N	Data source
African	0.925	0.075	0.850	0.150	0.000	246	*
African-American	0.893	0.107	0.787	0.213	0.000	61	*
European	0.883	0.117	0.778	0.208	0.013	379	*
American in Utah	0.888	0.112	0.788	0.200	0.012	85	*
American mixed	0.862	0.138	0.757	0.210	0.033	181	*
Mexican in Los Angels	0.803	0.197	0.667	0.273	0.061	66	*
Japanese	0.775	0.225	0.618	0.315	0.067	89	*
Japanese (lung cancer)	0.748	0.252	0.558	0.380	0.062	387	This study
Taiwanese	0.726	0.274	0.524	0.405	0.071	168	This study
Chinese in Beijing	0.722	0.278	0.515	0.412	0.072	97	*
Southern Han Chinese	0.710	0.290	0.500	0.420	0.080	100	*

N, the number of subjects.

* 1000 Genomes. http://browser.1000genomes.org/Homo_sapiens/Variation/Population?db=corer=2∶178129537–178130537;v = rs6721961;vdb = variation;vf = 4574214.

### SNP (c.–617C>A) in the *NRF2* Gene as a Biomarker for Prognosis of Lung Cancer

The *NRF2* gene is regarded as a double-edged sword. It plays an important role in protecting normal cells from external toxic challenges and oxidative stress, whereas it can also endow cancer cells resistance to anticancer drugs. Recently it has been reported that NRF2 contributes to the malignant phenotypes of cancer cells *in vitro*, including aggressive cell proliferation, drug resistance, and metabolic re-programming [8],[20]. Indeed, NRF2 activation is involved in the emergence of cancer resistance to various anticancer drugs by transcriptionally activating a battery of self-defense genes, such as those encoding antioxidant enzymes, phase II detoxifying enzymes, and ABC transporters [23]–[25]. ABCG2 is known to mediate the efflux of gefitinib (Iressa®) from cancer cells [39], and its expression is regulated by NRF2 [26] and the EGFR-tyrosine kinase cascade [40], [41].

As revealed in the Kaplan-Meier plot (Figure 2), lung cancer patients (both females and males) with homozygous SNP alleles (–617A/A) in the *NRF2* gene had markedly high overall survival. Univariate analysis showed a significant difference between the –617 A/A and –617 C/A groups in terms of overall survival (log-rank *P = *0.021). It is important to note that, except for one patient (case 5 in Table 5) who died because of primary pancreatic cancer, all of the adenocarcinoma patients with homozygous SNP alleles (–617A/A) in the *NRF2* gene survived over 1,000 days after surgical excision of the tumor that was followed up with neither adjuvant therapy nor chemotherapy, even when p-stage I patients were considered alone (Figure 2B). To our knowledge, this is the first report providing clinical evidence that homozygous SNP (–617A/A), as one of the intrinsic genetic polymorphisms in the *NRF2* gene, is associated with overall survival of lung cancer patients.

**Figure 3:**
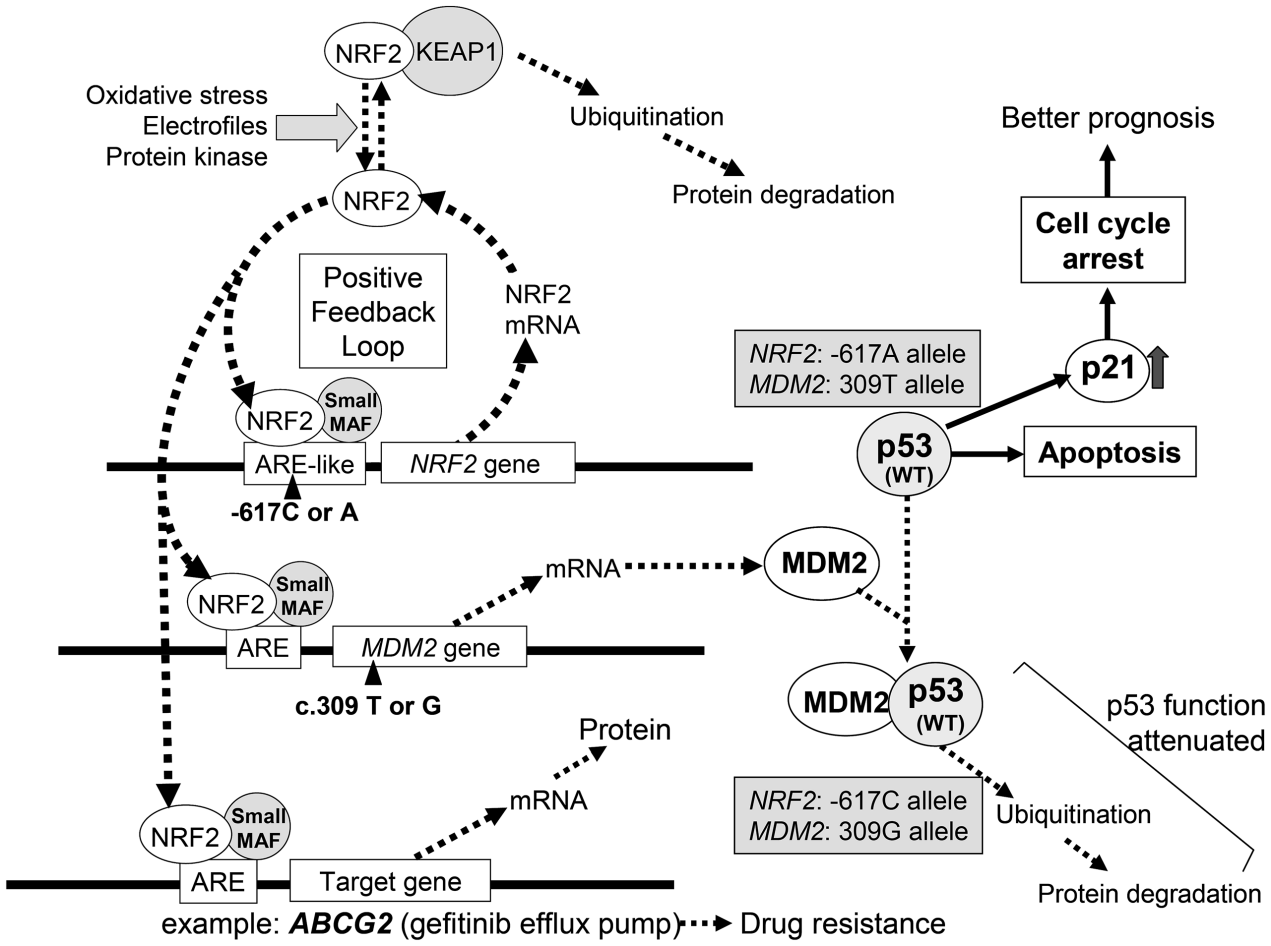
Schematic illustration showing the effect of *NRF2* SNP–617C>A and *MDM2* SNP c.309 T>G on the p53-mediated suppression of cancer cell proliferation and drug resistance. In response to oxidative stress, electrophiles challenge, or protein kinase-mediated phosphorylation (*e.g.*, via the PI3K-Akt pathway), the NRF2 protein is released from KEAP1 and then translocated into the nuclei. The SNP–617C>A in the ARE-like motif is considered to play a role in the positive feedback loop of transcriptional activation of the *NRF2* gene. The SNP homozygote (–617 A/A) significantly attenuates the positive feedback loop and also expression of NRF2-target genes, such as *MDM2* and *ABCG2*. In the case of *MDM2* gene expression, the SNP (c.309 T>G) in the first intron of the *MDM2* gene increases the binding affinity toward Sp1 and results in higher expression levels of MDM2 protein. MDM2 protein, thus highly expressed, binds to p53 (wild type; Wt) protein and leads to ubiquitination and proteasomal degradation of p53 (Wt) protein. Combination of the 309G (SNP) allele of the *MDM2* gene and the –617C (Wt) allele of the NRF2 gene may have negative impacts on p53 (Wt)-mediated tumor suppression. On the other hand, lung cancer patients harboring both the 309T (Wt) allele of the *MDM2* gene and the –617A (SNP) allele of the *NRF2* gene may have better prognosis due to the tumor suppressor function of p53 (Wt), such as apoptosis and p21^WAF1/cip1^-mediated cell cycle arrest. Expression of the *ABCG2* gene is known to be up-regulated by NRF2. Gefitinib, an inhibitor of EGFR tyrosine kinase, is extruded by ABCG2 out of cancer cells. Thus, NRF2-mediated induction of ABCG2 expression can confer cancer cells with acquired resistance to gefitinib and other anticancer drugs.

SNP –617C>A is considered to play a pivotal role in the positive feedback loop of transcriptional activation of the *NRF2* gene to regulate the NRF2 protein level (Figure 3). Since the SNP (c.–617A) in the ARE-like loci of the human *NRF2* gene decreases the binding affinity to the transcription factors of NRF2/small MAF [22], it is anticipated that the homozygote –617A/A significantly attenuates the positive feedback loop of transcriptional activation of the *NRF2* gene.

It has recently been reported that NRF2 regulates the basal expression of the murine double minute-2 (*Mdm2*) gene [42]. Since human MDM2 is an oncoprotein that binds to p53 protein and inactivates the tumor suppressor activity of p53 [43], NRF2 can indirectly contribute to p53-mediated cell cycle control and/or apoptosis [44]. One SNP in the *MDM2* promoter region, a T-to-G change at nucleotide c.309 (rs2279744) in the first intron, increases the binding affinity toward stimulatory protein 1 (Sp1) and results in higher expression levels of MDM2 protein [45]. This, in turn, attenuates the p53 tumor suppressor pathway and accelerates tumor formation in humans [45]. Asians, including Japanese, have higher frequencies of the 309G allele as compared with African-Americans and Caucasians [46]. It has been reported that this polymorphism in the *MDM2* gene is associated with the prognosis for several types of tumors, including lung cancer [47].

As demonstrated in Table 6, the 309G (SNP) allele frequency of the *MDM2* gene was markedly lower (0.333) in the genotype group of *NRF2* –617A/A, as compared with those observed in the genotype groups of *NRF2* –617C/C and –617C/A. It is suggested that lung cancer patients harboring both the 309T (WT) allele in the *MDM2* gene and the –617A allele in the *NRF2* gene have better prognosis owing to well-controlled tumor suppression via cell cycle arrest and/or apoptosis mediated by p53 (WT); refer to Figure 3. This may explain, in part, our finding that the lung cancer patients with homozygous SNP alleles (–617A/A) in the *NRF2* gene had markedly high overall survival rates (Figure 2).

In cancer tissues, somatic mutations take place frequently. In addition to the above-mentioned genetic polymorphisms as the “intrinsic” mechanism, mutations in the *KEAP1* and/or *NRF2* genes are the “acquired” mechanisms that lead to constitutive activation of NRF2. In fact, mutations in the *NRF2* and *KEAP1* genes have been found in carcinomas of the lung [12], breast [48], liver [49], and stomach [49]. Abnormalities in NRF2 activity were correlated with poor prognosis, when measured either as recurrence-free or overall 5-year survival. A recent immunohistochemical study has revealed that increased expression of NRF2 protein and decreased expression of KEAP1 protein are common abnormalities in NSCLC and are associated with poor prognosis [50]. Importantly, abnormal expression of NRF2 and KEAP1 proteins was more common than that of the corresponding gene mutaions [50], suggesting the involvement of other mechanisms such as intrinsic genetic polymorphisms of those genes. To bridge the gap between the homozygous SNP alleles (–617A/A) in the *NRF2* gene and the high overall survival of lung cancer patients shown in this study, we need to carry out further clinical follow-up studies with lung cancer patients (p-stages III and IV) who have been subjected to chemotherapeutic treatments. As exemplified in the present study, genetic polymorphisms/mutations and fine balances among NRF2, KEAP1, MDM2, p53, p21^WAF1/cip1^ and other genes are likely to contribute to the progression of cancer and, consequently, the prognosis of cancer patients.

### Development of a Rapid Genotyping Method for Personalized Cancer Therapy

One of the challenges in lung cancer management is to identify biomarkers for personalized cancer therapy. To effectively advance personalized medicine, cost-effective methods should be developed for genotyping. It would be desirable to include such information in each patient’s record as guidance for medial doctors to provide individualized treatment. In the present study, we have developed a rapid genetic testing method to elucidate the impact of genetic polymorphisms in the *NRF2* gene on the risk and survival of patients with primary lung cancer. The method enables the detection of genetic polymorphisms in target genes within 30 to 45 minutes under isothermal conditions that do not require DNA isolation and PCR amplification. Thus, this genotyping method would provide a simple and practical tool for personalized cancer therapy and assessment of prognosis.

## Materials and Methods

### Patients and Sample Collection

This clinical research was conducted according to the Declaration of Helsinki Principles. Under written informed consent, we collected blood samples from patients with primary lung cancer who received surgical operation at the Kanagawa Cancer Center. Protocols for sample collection, anonymity, storage, and transportation to RIKEN Yokohama Institute required for the present study were approved by both the Institutional Review Board of the Kanagawa Cancer Center Research Institute and the Research Ethics Committee at RIKEN Yokohama Institute. Procedures for analyzing the *HO-1* gene 5′-flanking region sequence as well as for genotyping the *NRF2*, *CYP2A6*, and *MDM2* genes in the genomic DNA samples were approved by the Research Ethics Committee at RIKEN Yokohama Institute.

The Taiwanese samples (N = 168) used in this study were randomly selected from the Han- Chinese Cell and Genome Bank in Taiwan described previously [51], in which more than 3,300 healthy controls were collected and randomly selected through registry.

### Preparation of Genomic DNA

Peripheral venous blood samples from lung cancer patients were collected into tubes containing Na_2_EDTA. Genomic DNA was extracted by the use of the QIAamp blood kit (QIAGEN K.K., Tokyo, Japan) according to the manufacturer’s instructions.

### Genotyping

Based on the SmartAmp method, rapid genotyping primers were developed for detecting both the SNP (c.–617C/A) in the ARE-like loci of the human *NRF2* gene (Figure 1) and the CYP2A6*4 genotype (whole gene deletion) [51]. We used exciton-controlled hybridization-sensitive fluorescent primers for optical detection of genotyping reactions (Figure 1). After genomic DNA was denatured at 98°C for 3 min, the genotyping reactions were allowed to proceed isothermally at 60°C for 60 min in a Mx3000P PCR system (Agilent Technologies, Santa Clara, CA, USA). The SNP c.309T>G in of the *MDM2* gene were detected by the Duplex SmartAmp method, as described previously [46].

### Analysis of Length Variability of (GT)_n_ Repeats in the *HO-1* Gene Promoter

The partial genomic DNA including (GT)_n_ repeats located in the 5′-flanking region of the *HO-1* gene was amplified by the polymerase chain reaction (PCR) [53], [54] with a fluorescence probe-labeled sense primer p1-s (5′-AGAGCCTGCAGCTTCTCAGA-3′) and an unlabeled anti-sense primer p1-as (5′-ACAAAGTCTGGCCATAGGAC-3′). These primers were designed according to the previously reported sequences [55]. The PCR cycle program of 95°C for 30 seconds, 63°C for 30 seconds, and 60°C for 30 seconds was carried out for a total of 30 cycles. The resulting PCR products were visualized by electrophoresis in 3% agarose gels containing ethidium bromide. The electrophoresis revealed two differently-sized PCR products attributable to two alleles with different (GT)_n_ repeat sequences in the *HO-1* gene. The (GT)_n_ repeats in the PCR products were analyzed with a laser-based automated DNA sequencer (ABI PRISM 3100 DNA Analyzer, Applied Biosystems Ltd., Tokyo, Japan).

### Analysis of Mutation Status in the *EGFR* Gene

DNA samples were isolated from frozen tissues or formalin-fixed and paraffin-embedded tissue sections that had been surgically excised from lung cancer loci. Epidermal growth factor receptor (*EGFR*) gene exons 19 and 21 were analyzed for their mutational status by the loop-hybrid mobility shift assay, a PCR-based heteroduplex analysis method, as described previously [56], [57].

### Statistical Analysis

The association of lung cancer with the allele frequency of the gene of interest was assessed by considering the confounding effects derived from known risk factors, such as age, gender, smoking history, and histopathology. After preliminary bivariate analysis by using Fisher’s exact test or χ^2^test, the multivariate logistic regression method was performed to estimate independent variables associated with the SNP homozygote (–617A/A) in the *NRF2* gene. Furthermore, the Kaplan-Meier method was used to estimate survival curves for overall survival. Log-rank tests were used to compare the survival curves of patients in different *NRF2* subgroups. The statistical significance of all the data was tested by the analysis of variance. We performed statistical analysis with the SPSS statistics program (v.19.0; SPSS Inc., Chicago, USA). Values of *P*<0.05 were considered to indicate statistical significance.

## Supporting Information

Table S1 **Classification of female adenocarcinoma patients with respect to genotypes of** ***NRF2*** **and** ***MDM2*** **genes.**
(DOC)
