# Supplementary material for: SNP (–617C>A) in ARE-Like Loci of the NRF2 Gene: A New Biomarker for Prognosis of Lung Adenocarcinoma in Japanese Non-Smoking Women
Source: PLoS One. 2013 Sep 11;8(9):e73794. doi: 10.1371/journal.pone.0073794 (PMC3770684; doi:10.1371/journal.pone.0073794)
Supplement: Table S1 — Classification of female adenocarcinoma patients with respect to genotypes of NRF2 and MDM2 genes. (DOC) [file pone.0073794.s001.doc]

**Supplementary Table S1**

**Classification of female adenocarcinoma patients with respect to genotypes of *NRF2* and *MDM2* genes.**

|  |  | NRF2 (-617) |  |
| --- | --- | --- | --- |
|  | C/C | C/A | A/A |
| Patients (N) | 78 | 53 | 16 |
|  |  |  |  |
| MDM2 (c.309) | N (%) | N (%) | N (%) |
| T/T | 14 (17.9) | 13 (24.5) | 8 (50.0) |
| T/G | 34 (43.6) | 21 (39.6) | 7 (43.8) |
| G/G | 30 (38.5) | 19 (35.9) | 1 ( 6.2) |

N, the number of patients; % in parentheses
